# Quality assessment of higher resolution images and videos with remote testing

**DOI:** 10.1007/s41233-023-00055-6

**Published:** 2023-04-13

**Authors:** Steve Göring, Rakesh Rao Ramachandra Rao, Alexander Raake

**Affiliations:** Audiovisual Technology Group, TU Ilmenau, Ehrenbergstraße 29, Ilmenau, 98693 Thuringia Germany

**Keywords:** Image quality assessment, Video quality assessment, Remote testing, Crowdtesting, HTTP-based adaptive streaming

## Abstract

In many research fields, human-annotated data plays an important role as it is used to accomplish a multitude of tasks. One such example is in the field of multimedia quality assessment where subjective annotations can be used to train or evaluate quality prediction models. Lab-based tests could be one approach to get such quality annotations. They are usually performed in well-defined and controlled environments to ensure high reliability. However, this high reliability comes at a cost of higher time consumption and costs incurred. To mitigate this, crowd or online tests could be used. Usually, online tests cover a wider range of end devices, environmental conditions, or participants, which may have an impact on the ratings. To verify whether such online tests can be used for visual quality assessment, we designed three online tests. These online tests are based on previously conducted lab tests as this enables comparison of the results of both test paradigms. Our focus is on the quality assessment of high-resolution images and videos. The online tests use AVrate Voyager, which is a publicly accessible framework for online tests. To transform the lab tests into online tests, dedicated adaptations in the test methodologies are required. The considered modifications are, for example, a patch-based or centre cropping of the images and videos, or a randomly sub-sampling of the to-be-rated stimuli. Based on the analysis of the test results in terms of correlation and SOS analysis it is shown that online tests can be used as a reliable replacement for lab tests albeit with some limitations. These limitations relate to, e.g., lack of appropriate display devices, limitation of web technologies, and modern browsers considering support for different video codecs and formats.

## Introduction

Multimedia quality assessment is one example of several research fields relying on data that has been annotated by humans. For such quality assessment, subjective studies are considered a gold standard. Traditionally subjective tests are conducted in a controlled lab environment following standard recommendations such as BT.500-13 [43] to assess the perceptual image and video quality. In addition, a given procedure and additional checks (e.g. pre-questionnaires, vision tests, colour blindness tests) can be used to ensure that highly reliable labels for quality assessment are gathered. However, lab tests are both time-consuming and expensive. Furthermore, conducting subjective tests in such lab settings may not always be possible due to various reasons, for example, a research group may not have access to those standardized test rooms and equipment, or it may not be allowed to perform lab tests due to unforeseen circumstances, e.g. COVID-19 pandemic. As an often more practical alternative to subjective quality ratings, the assessment of video systems and services is often performed using so-called objective quality models. These are trained to predict subjective ratings from human subjects and can be applied for encoding evaluation or service delivery monitoring [69, 81]. A major requirement to train and validate such models is that they have access to a wide range of realistic subjectively rated encoded videos or data annotated accordingly.

To address both the problems of expensive and time-consuming lab tests and the need for a large amount of ground-truth data for the development of image and video quality models, as in various other fields of research, crowd or remote testing is used as an alternative for multimedia quality assessment [16, 33, 82, 99]. Conducting video quality assessment in the crowd may have, with the right choice of the panel, the additional benefit of a large and diverse, possibly international, geographically distributed set of users in realistic settings [32]. On the other hand, it also means that tests are no longer performed in controlled settings conforming to standard recommendations. Several comparisons between lab and crowd tests show a good correlation between the results, similar to usual inter-lab test correlations [50]. To ensure reliability in crowdsourcing experiments, studies have been conducted to analyse and discuss conceptual, technical, motivational, and reliability challenges [48], and to compile a set of best practices for crowdsourcing QoE testing [32].

In general, such crowd, online or remote testing approaches have been shown to be efficient in assessing perceived image and video quality. However, most of the studies were limited to lower-resolution images and videos, and hence, there is a lack of investigations on the perceived quality of high-resolution images and video in a non-lab setting. The use case of quality assessment of high-resolution images and videos is important due to the widespread creation, upload, and viewing of this content on different platforms such as Flickr, and Instagram for images, and video streaming services such as Netflix [68], YouTube, Amazon Prime, and others. With this focus, the following two research questions can be identified and will be addressed in the remainder of the paper.

- How can crowd or remote testing be applied to the quality assessment of high-resolution images and videos?
- How reliable are the results of the crowd or remote tests in comparison with traditionally conducted lab tests?

Important for the research questions to be answered are several aspects, for instance, the crowd and lab tests are similar in their corresponding design, thus sharing the same stimuli, to enable a good comparison. And moreover, remote or crowd tests differ from usually conducted lab tests regarding the duration or diversity of devices or participants [36], which results in the requirement to adapt the test design to the crowd scenario. For example, in a lab test, high-quality and standardized equipment could be used, whereas, in the case of the crowd or remote tests, the devices of the participants can only be employed for the given tasks, which may not follow the most recent technology trends. Here, the key important aspect is, that we target higher-resolution images and videos, where it should be noted, that not all participants may have appropriate displays and computers to present the stimuli.

To tackle, the display resolution problem and hence address the first research question, in this paper, we propose and evaluate an approach for using crowdsourcing to assess the perceived quality of higher resolution images and videos up to a resolution of 4K/UHD-1. For images, a patch-based approach using 1080*p* patches is used for quality assessment in the crowd. For the video quality evaluation, the approach is based on using a pre-defined crop cut-out from the centre of the original 2160*p* video as the stimulus presented to the participants in the web-based crowdsourcing platform. This proposed approach is tested for both short-term video quality assessment and the overall quality assessment of a HAS session. Both, the patching and centre cropping approaches, which are used in this work, are similar to the patch-based approach used by Bosse et al. for the evaluation of perceived image quality [8] and by Göring et al. [21] and Keimel et al. [49], who used a centre crop approach for full-reference and no-reference video quality model evaluation respectively. It is shown in [21] that centre-cropped variants of videos can be used to objectively assess quality with only a marginal decrease in prediction performance as compared to the performance when using the full-frame for quality prediction.

The second research question is tackled by using corresponding lab tests and then comparing the results of the lab and crowd tests. The reliability of the patch-based approach for quality assessment of high-resolution images is investigated by comparing a lab and a corresponding online test. For short-term video quality assessment, the proposed crowd-testing procedure is validated by using the lab test results of test_1 of the publicly available AVT-VQDB-UHD-1 [86] dataset containing videos of up to a resolution of UHD-1. The short-term videos have a duration of $10\,s$ and degradations considering various encoding parameters (codecs, bitrate, and resolution). Similarly, the applicability of the crowd paradigm for the overall quality assessment of a HAS session is tested by comparing the results of the crowd test with the corresponding lab test, which has been conducted as part of the *P.NATS Phase 2* competition [81]. The long-term video quality tests consist of videos of $2\,min$ duration with degradations such as video quality switches and stalling events.

The paper is organized as follows. In “Related work” section a brief overview of quality assessment and crowd or remote testing for images and videos is presented. Afterward, a description of our developed online testing framework AVrate Voyager is presented in “Remote testing framework” section. This section is followed by “Image quality assessment” section which compares a lab and remote test considering image quality with a focus on high resolutions and the required adaptations of the remote test. Similarly, in “Short-term high resolution video quality assessment” section and “Long-term audiovisual quality assessment” section lab and remote tests for the quality assessment of higher-resolution short-term and long-term videos are compared. Finally, the paper ends with a discussion of the results and a conclusion with an outline of future work in “Discussion and conclusion” section.

## Related work

In the following section, a brief review of image and video quality assessment for higher resolutions is presented. The focus of this section is on the methods available for quality assessment of images and videos using crowdsourcing and whether crowdsourcing or online tests are applicable for this use case. To implement crowd or remote tests it is usually required to use a web-based system to, for example, show the stimuli and collect the ratings. For this reason, an overview of existing remote testing frameworks is outlined and discussed which are suitable for image and video quality evaluation considering higher-resolution content.

### High resolution image quality assessment

Newer image codecs and methods have been developed for higher-resolution images, e.g., AVIF [74] or HEIF [53, 72]. Therefore, it is also important to assess the effect of these new codecs on the quality of such high-resolution images. Here, a limiting factor is suitable datasets, because most published data either use lower resolutions or include only JPEG compressed images, for example, the Tampere Image Dataset 2013 [79]. However, it is shown in [23] that video compression methods applied to images can outperform classical state-of-the-art image compression in comparison to, for example, JPEG.

Recent developments, such as the JPEG-AI competition,^1^ focus on image compression methods that are learning-based and suitable for higher-resolution images. Those learning-based methods can be implemented using DNNs [10, 62, 116] or use hybrid approaches that rely on traditional methods combined with neural networks for image enhancement [56]. An example of such a hybrid variant is proposed by Lee et al. [56]. Lee et al. [56] use VVC [42], a recently published video encoder, to compress images and later perform image enhancement using a deep neural network.

The current most popular web image formats are JPEG, GIF, and PNG [13] respectively. Newer formats are WebP [20], BPG [6], HEIF [53, 72] or AVIF [74]. All four new formats have in common that they rely on video compression algorithms, e.g., WebP is based on VP8, BPG is based on a modified HEVC variant, HEIF uses HEVC, and AVIF is based on AV1. The trend to use video coding approaches for images leads to the question of whether they can outperform traditional methods in compression efficiency and quality. However, only a few published studies compare newer developed methods. For example, in [53], it is shown that HEVC/H.265 is able to save bitrate while keeping the same quality in comparison with JPEG. The evaluation was performed using 14 high-resolution images (height/width of maximum 4096 pixels). Moreover, other studies confirm that HEVC’s intra-frame coding is a well-suitable still image compression approach [71]. Besides HEVC, VP9, and VP8, AV1 is another promising video codec, however, there are only a few studies available comparing still image compression of AV1 or the AVIF format [5]. In [14], the still image compression performance of the Daala video codec is analysed. The evaluation of Daala’s compression ability is performed using 8 images up to Full-HD resolution. However, the Daala codec development is mostly subsumed in AV1. Barman et al. [5] compares JPEG, WebP, JPEG-2000, HEVC, and AVIF using objective quality metrics such as VMAF, SSIM, VIF, and PSNR. The evaluation is performed using three different datasets, consisting of images with a resolution of $2040\times 1346$ and $1920\times 1080$ thus approximately 2K and Full-HD resolutions. Based on the results, it can be concluded that AVIF outperforms other methods considering the quality and bitrate savings. However, it should be mentioned that there are no high-resolution images (higher than 2K) included in the evaluation. For this reason, another evaluation is required.

Moreover, most of the image compression benchmarks or comparisons are based on PSNR [1] or other objective metrics, while it is already shown that there is only a medium or low correlation with subjective scores [79, 102]. For example, in the Tampere Image Database 2013 (TID2013) [79] PSNR has the lowest Pearson correlation to subjective scores when only JPEG compression artefacts are considered. The TID2013 consists of medium-resolution images and includes different distortions, e.g., noise. Furthermore, most of the recently published databases focus on medium-resolution images, e.g., the KonIQ-10k Dataset [37], KADID-10k [60], or the LIVE In the Wild Image Quality Challenge Database [18]. Such datasets target user-generated content, include a larger number of images and the quality ratings are gathered using crowdsourcing studies. Most of these datasets can be used to train deep neural networks for image quality prediction, as is also shown in [22, 37, 59]. On the other side, especially for videos, there are datasets available focusing on higher resolutions. In addition, also video quality models for higher-resolutions show high correlation with subjective scores, e.g., Netflix’s VMAF [61, 69] for UHD-1/4K video contents [25, 26, 86], or the recently standardized P.1204 series [81, 85].

As mentioned before, image quality or general QoE tests can also be conducted using crowdsourcing, remote or online tests [35, 38, 39, 65, 92]. However, a general drawback of such online testing is that there is less control regarding environmental factors, the used setup to perform the test, general distractions, and more [36]. For example, it can be assumed that usual remote participants do not have a high-resolution display with a powerful PC to play uncompressed videos or to show high-resolution images. On the other side, crowdsourcing or remote tests include more variation in terms of the participants and it could be assumed that their used environment and setup are more realistic. Further, crowdsourcing or remote tests require more effort in designing, the inclusion of hidden conditions as checks, or reduced overall duration [36]. Here, a linking of standardized methods and crowdsourcing or remote approaches can be used to evaluate the reliability of such test paradigms. For example, in [65], such an evaluation for speech quality assessment is performed. Naderi et al. [65] show that standardized methods and crowdsourcing yield comparable results. Moreover, crowdsourcing has also been widely used in the perceptual assessment of image quality and in creating large image datasets annotated with human ratings. For example, Ghadiyaram et al. [19] designed and created the “LIVE in the Wild” image quality challenge database consisting of 1162 images rated by over 8100 unique observers. In addition, Hosu et al. [37] created an image database consisting of 10073 images scored in terms of quality by 1459 crowd users, and furthermore an extended version KonIQ++ [107] with included annotations regarding distortions. On the other hand, as stated above, Bosse et al. [8] investigated the feasibility of patch-based image quality assessment and found that humans can evaluate perceived quality on a patch size of 128 $\times$ 128 pixels from a source image of 512 $\times$ 512 pixels.

The crowdsourcing or remote paradigm for quality assessment of higher-resolution images is still challenging. Most of the image quality datasets focus on lower or medium-resolution images. While in addition, most of the aforementioned studies do not include more recently developed image compression methods.

### Video quality assessment for UHD-1/4K

Similar to image quality assessment, video quality is usually evaluated in traditionally conducted lab tests, especially in the case of higher resolutions such as UHD-1/4K or even UHD-2/8K. There are various examples of quality evaluation for videos reported in the literature, e.g., [4, 11, 12, 31, 46, 47, 58, 86, 111, 115]. All the mentioned studies have in common that they are conducted in controlled lab environments. Moreover, to analyse the differences between Full-HD and UHD, and if users are able to perceive a difference, Berger et al. [7] present results of a lab test comparing the perceived quality of transmitting UHD-1 content compared to Full-HD content at the same bitrate, encoded with HEVC. In addition, Van Wallendael et al. [111] performed a similar lab test, where 4K and HD resolutions were compared. They also arrive at a similar conclusion as [7], namely that the perceptibility of a 4K advantage is highly content-dependent. In [27], Göring et al. developed an automated system to predict whether there is a benefit of using UHD over HD or not. Here, nearly 50% of their analysed uncompressed source videos will not have any perceivable benefit when shown in UHD. For the training and evaluation of this system, two tests have been conducted in a lab setup. Moreover, Rao et al. [86] performed four subjective tests considering UHD-1/4K video quality. The focus of the lab tests was different encoding settings and video encoders, the data is publicly available.

For the assessment of video quality, Hoßfeld et al. [33] propose a generic subjective QoE assessment methodology for multimedia applications based on crowdsourcing. They conclude that crowdsourcing is a highly effective method not only for QoE assessment of online videos but also for other current and future internet applications. A study on the usage of crowdsourcing for subjective quality assessment in the HTTP-based adaptive streaming (HAS) context was conducted by Shahid et al. [100]. Here, the results of the crowdsourcing test showed a strong correlation with the corresponding lab test. Similarly, Rainer et al. [82] conducted a crowdsourcing study in the HAS context with the objective of comparing the QoE performance of different HAS-based web clients namely, YouTube, DASH-JS, and dash.js. The study concludes that the delivered representation bitrate and the number of stalls are the main influencing factors of QoE, as can also be confirmed by lab studies [93].

In addition, crowdsourcing has been used to create large video datasets annotated with human ratings. A few examples are the Konvid-1K database by Hosu et al. [39] which consists of 1200 public-domain video sequences sampled from YFCC100m [109], containing a very small number of high-quality videos. In addition, the KonViD-150k [29, 30] has been published, including 150k videos 720*p* videos similar to the Konvid-1K dataset. Furthermore, the LIVE-VQC dataset is another dataset that has been created by Sinno et al. [105] and consists of 585 videos with 240 recorded human ratings per video.

Notably, Seufert et al. [99] conducted a crowdsourcing study to test the limits of crowdsourced subjective video quality testing. They investigated the extreme case of presenting only a single test condition with a stimulus duration of 10 s to each subject (i.e. fully corresponding to a between-subjects test design) and the possibility of using such a simple “one-shot” design with a large number of subjects instead of using sophisticated test designs in crowdsourcing. The results suggest that when training effects are negligible, the “one-shot” design seems to be applicable. In this study, source videos of 1080*p* were downscaled to 576*p* to meet the possibly low internet connections of the crowd users. So the overall video resolution was limited, and hence considering higher-resolution videos is still challenging in such a crowd scenario.

Recently, Uhrina et al. [110] investigated the feasibility of using an unpaid crowdsourcing approach as a replacement for lab-based subjective testing and reported a correlation of more than 0.92 between lab and crowd tests. The most notable aspect of this study is the usage of videos of resolutions up to Full-HD. Moreover, in [64] Full-HD videos are evaluated using crowdsourcing, overall the results indicate a similar performance to a lab test. A similar correlation between lab and crowd tests has also been reported by Saupe et al. [97] in their study of using crowdsourcing for subjective video quality assessment using the paired comparison approach.

In addition to multimedia quality assessment, crowdsourcing or remote testing has been used in other multimedia applications such as image annotation [73, 88], video summarization [106, 108], speech quality assessment [65], 3D objects [67], point clouds [75], and visual attention [55]. Important to mention here, is that to evaluate the reliability of crowdsourcing studies, in the best case a comparison of the crowd or remote results and lab tests is performed, e.g. as done in [50, 67, 75, 96, 100].

### Testing frameworks

Performing tests with humans being involved is a crucial part of several research fields, e.g. quality assessment of multimedia contents, i.e. audio, video, or images, to improve compression or analyse perception [80]. A commonly established method to conduct quality evaluation is to perform a lab test, where a participant is asked to rate a specifically presented and prepared stimuli in a controlled environment, following recommendations such as ITU-R BT.500-13 [43], ITU-T Rec. P.913 [41], or ITU-T Rec. P. 910 [89]. On the other side, next to the well-established lab tests, crowdsourcing tests are increasing in popularity for such perception tests. For example, it was already shown that crowd sourcing can be used for audio [65, 66], video [16, 33, 82, 87, 100, 105] and image quality assessment [18, 37, 39, 90].

The usual approach for crowd tests is to recruit participants from a large anonymous crowd, and each participant takes part in a small study [15, 36]. The study is usually implemented in an online test, while all data is collected and stored. To implement such online tests typical crowdsourcing providers offer their own platforms and frameworks. However, such platforms are usually optimized for a micro-tasking approach and are limited in their flexibility and adaptability. For this reason, a specialized online test software is required that can be adapted to different test designs easily.

For example, one tool to implement questionnaires is The Fragebogen.^2^ The tool provides a common framework based on JavaScript and HTML to implement offline or online questionnaires with pre-defined elements. It would be possible to include video or audio in the questionnaire itself, however, the overall framework targets text-based surveys.

Another tool for online studies [50] is QualityCrowd2^3^. Keimel et al. [50] propose a PHP based framework to perform subjective video quality assessment. They show that the crowd approach produces similar reliable results compared to controlled lab tests. The overall test can be included in Amazon Mechanical Turk, a micro-tasking-based crowdsourcing platform. QualityCrowd2 is an extension of the published QualityCrowd which has been described in the paper [50] However, the tool seems to be outdated because only minor changes have been done within the last years when checking out the GitHub page. Also, the QualityCrowd and QualityCrowd2 systems use Adobe Flash Player to play out videos, which is deprecated^4^ and replaced by HTML5 technology. A similar framework is WEST,^5^ it also uses PHP and targets mobile devices, however, it also seems to be outdated and less usable. In addition, Naderi et al. [64] propose a framework for a crowd micro-tasking approach (using e.g. Amazon Mechanical Turk) following ITU-T Rec. P. 910 [89].

Similar to online test frameworks, software for lab tests is available. For example, VQEGplayer^6^ [9] can be used for tests using windows. Furthermore, AVRate^7^ [54] is another windows only lab test software. AVRate can be used for audio, video, and audiovisual user tests. It can handle various video players and can be configured using XML for different rating scales. As an extension and re-release AVRateNG^8^ has been proposed by us. AVRateNG is similar to AVRate, though, it uses web technology to be scalable and operating system independent.

The main purpose of AVRateNG during its development was to perform high-resolution video-quality lab tests. Therefore, we conducted several UHD-1/4K quality tests with AVRateNG as shown in [25, 28, 52, 81, 83, 84, 86]. The usual AVRateNG procedure is to use a command-line player, e.g., mpv,^9^ ffmpeg/ffplay^10^ or similar depending on the test design, to play out the given stimuli. AVRateNG handles the presentation, collection of ratings, and playout of the media files using the configured command-line player. For the presentation of the rating scheme and questionnaire, AVRateNG uses web technology in a client–server approach, i.e., Python 3, Bootstrap, and Bottle with a focus on a local setup. The rating scheme can be changed, and all ratings are collected in the database of AVRateNG and evaluated further. In a similar approach, AVRateNG has been used in the studies by Pinson [77] and Ashimov et al. [2], here only the stimuli, questionnaire, and video conditions have been changed.

AVRateNG can also be used to just collect answers for a questionnaire, as it is shown in several studies by Singla et al. [103, 104] in the context of VR Video quality evaluation and simulator sickness. Additionally, AVRateNG can be used for the evaluation of specific aspects of music perception [51] in combination with an automated hardware setup for mobile end devices.

It is not a simple task to provide an overall generic framework for all subjective tests, for this reason even specialized software is required. For example, in the case of virtual reality to record the user’s head rotations while playing out a 360$^\circ$ video AVTrack360^11^ [17] has been proposed. Here, the general architecture of virtual reality applications like e.g. SteamVR makes it hard to use a web-based approach. Another problem, in this case, are questionnaires, for this reason, Regal et al. propose VRate [91], which adds a questionnaire inside the virtual environment.

Other specialized, and even web-based solutions are available, e.g., webMUSHRA^12^ [98] that specifically addresses listening tests for audio quality assessment using the MUSHRA test paradigm.

### Challenges

As mentioned before, visual quality assessment for videos or images is still widely performed in controlled lab environments. Higher-resolution images and videos are often used, and compression methods have been improved to reduce the required bandwidth for the final transmission. There are cases, where crowdsourcing is used to evaluate the quality, however, those studies usually target lower-resolution videos or images. For video quality evaluation most research was focused on resolution up to Full-HD, due to issues of controlling the display device, low bandwidth connections of crowd users, etc. There is a clear lack of crowdsourcing methods and also studies for quality assessment of high-quality/-resolution videos or images.

For this reason, in this paper, we propose in the following sections approaches for crowdsourcing-based image and video quality assessment considering high-quality and higher resolutions. Moreover, there are several frameworks for various test approaches published and some are publicly accessible. Each of the described approaches has its drawbacks, e.g., some are outdated, some are specific to video, some are only for audio, or target different use cases. To bypass the aforementioned limitations of remote rating frameworks, we developed our own rating framework, which is based on our lab-based rating software AVRateNG [3]. In the next section, we describe the test framework and the conducted studies for image and video quality assessment in detail.

## Remote testing framework

In the following, a brief description of the developed remote testing framework, which is called AVrate Voyager [24] is provided. The online or crowd tests, which will be described in the next Sections, have been conducted using this framework with minor adjustments. In general, the framework consists of several components and steps, and it is publicly available.^13^ It can be used to carry out remote tests, crowd tests, or other online tests. A detailed overview of the AVrate Voyager framework with screenshots of the UI and possible test instances can be found in [24].

AVrate Voyager uses scalable technology (Python 3, Docker, and HTML 5) to enable a unified and future-proof rendering on all possible end devices and an easy deployment. A web browser (e.g. Mozilla Firefox, Google Chrome) is used to start the test process from a user’s perspective. Furthermore, the application must be running on a web server, e.g. using docker.

The gathered data, e.g., quality annotations for images or videos, is stored in a SQLite 3 database. The final ratings can be either read directly from the database file or exported to CSV using the provided scripts of AVrate Voyager. Usually, the data covers the ratings, questionnaire, feedback, and user-specific information (e.g. the used browser, and the window dimensions) in an anonymous form. The overall configuration of AVrate Voyager is done using a global JSON file (e.g. to change the rating scheme), storing the stimuli files in specific folders, and adapting the provided templates to the needs of the test.

**Fig. 1:**
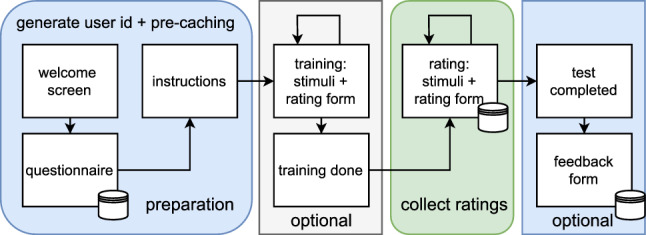
Test procedure implemented in the AVrate Voyager framework [24]

A typical test with the corresponding steps is shown in Fig. 1. A user starts with the “welcome screen”, here a general overview and explanation of the test are given. After this screen, the questionnaire is shown. In this step, the user id and the user’s individual stimuli list are generated, and in addition, all stimuli for the user are pre-cached using HTML5’s pre-fetching. The pre-caching is done to ensure that the stimuli files are fully or at least partially loaded. Moreover, depending on the configuration a user may get a fully random selection of all stimuli or only of a subset. The provided questionnaire is generic and can be adapted to the specific test.

After the questionnaire is filled, the user submits the results, and everything is stored in the database. Then the introduction follows, here specific explanations of the used rating scheme and guidance for the test can be placed. In addition, a check for the used window size and height (height of at least 600 px) is performed. The user is asked to maximize the browser window, otherwise the test cannot be continued, moreover landscape mode is preferred. Zoom and font settings are taking into account, however the final presentation of images and videos is fixed to a given resolution of the stimuli. Devices with a resolution that is too low are invalid, this check can be adjusted in one of the templates. After the instructions are completed, the training may start. The training is optional and will be just performed when media files (video, audio, or images) are stored in the ’train’ folder of AVrate Voyager. No ratings of the training phase are collected.

Subsequential to the training a small notification screen for the completion of the training part is shown, and the rating procedure is started. A rating screen consists of two generic elements, one is the presentation of the stimuli and the other one is the rating scheme. Both can be configured separately. For the rating scheme, currently ’Absolute Category Rating’ (ACR), ’Sliders’, and ’Labels’ are implemented. The provided templates are documented and can be easily extended. The stimuli presentation template is realized in a generic way and may need adaptation to the corresponding test. Similar to the rating scheme, the part is implemented as a template and can be adapted. The stimuli template is currently able to handle image, video, and audio files employing the HTML5 standard for multimedia output. It is important to check that the media format is compatible with the majority of browsers such as Mozilla Firefox or Google Chrome. For audio, FLAC is a possible lossless codec that is supported by most web browsers. In some cases of tests, it may be required to select a visually lossless approach of encoding to encode the videos to the final presentation format. For example, we figured out that H.264 with 4:2:0 8 bit (some browsers do not support other settings) and a CRF (Constant Rate Factor)-based encoding of 22–24 was suitable up to Full-HD resolution in previous video test runs. Video can be played in full-screen or in window mode, the template provides functionality for both cases. AVrate Voyager checks whether the multimedia stimuli file is fully loaded, e.g., to avoid stalling in case of video playback. Besides the rating itself, AVrate Voyager also stores the window height, width, and in the case of video or audio how often the corresponding media file was played. The template includes checks, that the stimuli have been played and that a rating has been performed. When all stimuli are rated, a final screen with a feedback form is shown. The user may add there some feedback for the test or just general comments. In each of the specific steps, some validation checks are performed in the back-end system, while some are handled using client-side cookies.

## Image quality assessment

In the following section, we describe in detail the design of the lab and online test for image quality. We start with the dataset, which uses UHD-1/4K video frames as a basis, the applied encoding scheme, and the selection of the used images for both of the tests. Afterwards, we describe the implementation of the lab and online test and compare both test paradigms, considering the required modifications. The scripts and data needed to reproduce the evaluation and results are publicly available.^14^

### Dataset and processing pipeline

**Fig. 2:**
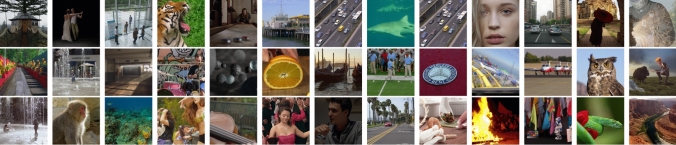
Overview of the used uncompressed and centre cropped source UHD-1/4K frames for the image quality evaluation

**Fig. 3:**
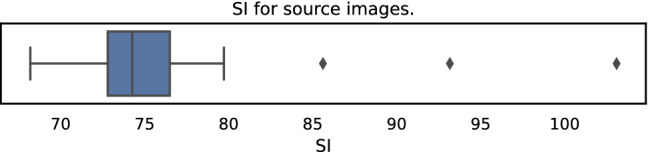
Barplot of SI for all used images

Typical high-resolution images have a larger resolution than Full-HD. As a starting point to analyse the quality of such images, UHD-1/4K video frames can be used and are widely accessible. In total 39 different single UHD-1/4K frames have been extracted from several uncompressed UHD-1/4K videos covering a wide range of realistic videos. The source videos were available in a 4:2:2 or 4:2:0 chroma sub-sampled 10 bits lossless video format. Subsequently, all frames are centre cropped to ensure that they have the same width and height of 2160 pixels. In Fig. 2 all used source images are shown, the selection is based on several different genres such as animated content, short movies, or documentaries to cover a wide range of realistic images. Furthermore in Fig. 3 we calculated Spatial Information (SI).^15^ It is visible, that the images cover a wide range of SI.

**Fig. 4:**
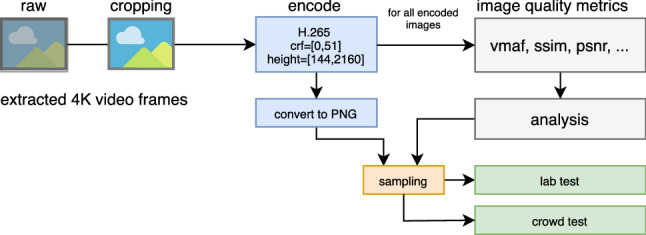
Image processing pipeline, starting from extracted raw 4K video frames, to cropping, encoding and designing subjective tests based on sampling

Further, all extracted single frames were encoded with H.265 using FFmpeg 4.1 with various resolutions into 246, 126 individual compressed images. H.265 was selected because it was already reported that it outperforms JPEG [23]. The general processing pipeline is shown in Fig. 4. Each image originating from a 4K video, is centre cropped to obtain images with $2160 \times 2160$ pixels. These images are then encoded with several settings. The encoding resolutions vary with a *height*/*width* in the range of $[144,\ldots , 2160]$ with a step size of 16 pixels. The specific small step size is selected to further analyse the impact of up-scaling algorithms on image quality. As encoding, a CRF based 1-pass scheme is used, here the value for CRF is varied within the range of $[0,\ldots , 51]$ with a step size of 1.

Afterwards, for all encoded images, several traditional objective image quality metrics were calculated. For all metric calculations, the VMAF tool is used, thus also a VMAF score is estimated. Even though VMAF is designed for video quality analysis, it is also suitable for images [5] and for higher-resolutions, e.g., for 4K video [26, 68, 70, 86]. In the case of images, it can be assumed that it is a still image video and the motion estimation feature can be neglected because it also has a generally lower impact on the estimated quality scores that underlay the VMAF calculation. This can be concluded by the low prediction performance of VMAF in the case of framerate variations for videos [86].

As next, some initial analysis of the extracted VMAF scores is performed. This analysis forms the base of data sampling to design the lab and online tests.

### Analysis of objective scores

**Fig. 5:**
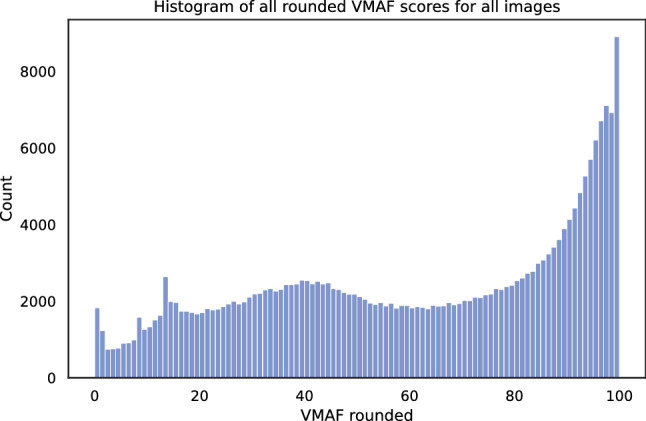
Histogram of rounded VMAF scores for all 246, 126 encoded images

**Fig. 6:**
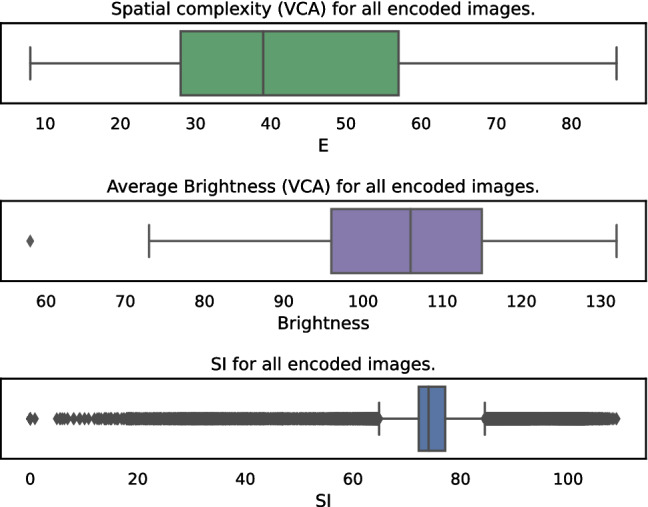
VCA (spatial complexity and brightness) and SI analysis of all encoded images

In Fig. 5 the distribution of all rounded VMAF scores for all encoded images is shown. Especially in the high-quality range, starting from a VMAF score of 85, it is visible that more often a similar quality score is reached. This leads to the conclusion that high-quality scores can be reached with several encoding settings. Moreover, in the following VMAF will be used as a criterion for the data sampling. However, to give a proper overview of the range, we further calculated SI and VCA [63] (Spatial Complexity and Brightness) for all encoded images. The results are summarized in Fig. 6 and show that in all three features a wide range is covered.

### Data sampling

To verify the suitability of VMAF and the compression performance of H.265, subjective tests can be used. However, some sampling of the encoded images is required, because it is otherwise not possible to perform a lab test, as every participant would need to rate all images to get mean opinion scores (MOS) for all images. For this reason, a first selection is performed. This selection uses one representative image for each rounded VMAF score $\in [0,100]$. Other sampling strategies would be possible, however, we selected VMAF because already showed a good performance for image quality prediction [5] and SI may be limited as a complexity measure, see also [94], and VCA [63] was not yet published as the sampling was performed. A criterion to sample representative images for each corresponding rounded VMAF score is needed. The first sub-sampling uses for each source image and for each rounded VMAF score the following approach. First, a selection was performed on images that have a lower *height* than the mean *height* of all images in the current rounded VMAF score group. Next, only CRF values lower than the mean CRF and larger than the median CRF of the remaining images are considered. Afterwards, the representative image was selected by the maximum remaining *height*. This ensures a deterministic sampling and based on the VMAF scores all images were similar in each of the groups, thus even different samplings would result in similar images.

Using the described approach of sampling it is possible to reduce the number of encoded images to approximately 100 stimuli per source image, in the remaining referred to as $ICF_{100}$. Here, it should be mentioned that some source images do not cover the full range of possible VMAF scores using the described encoding approach, e.g., some images show no changes in lower ranges due to the high spatial complexity of the source images. However, the mentioned sampling still creates for all source images in total approximately 3900 different distorted images. Because 3900 images are still not feasible for a test, a second sampling step was required to select suitable images for a lab test, in the following referred to as $ICF_{test}$.

Here, in the first step, for each image, the rounded VMAF scores are transformed linearly to [1, 5]-scaled MOS. Afterwards, each MOS is rounded to the next integer. For each source image, a selection is performed in the following way. For each rounded MOS two encoded images are randomly selected for the test. It should be mentioned that some source images have only one encoded image for a specific rounded MOS value, thus in these cases, only one image can be used in the resulting test. The second sampling step resulted in a total number of approximately 8 to 10 stimuli for each source image that is used in the final lab test. As a result, the overall test consists of 371 stimuli shown to the participants.

### Lab test for image quality

Using the images of the dataset $ICF_{test}$, a lab test was conducted. To enable high reliability of results and further reproducibility, the subjective test was implemented in a standardized lab environment as recommended in ITU-T P.910 [89] and ITU-R BT.500-13 [43]. The image stimuli were presented using a 4K screen (55 inches 4K LG OLED55C7D) with a viewing distance of approximately 1.5 to 1.6 times the height of the screen, as recommended in ITU-R Rec. BT.500-13 [43]. Before a participant rated the stimuli, a vision test (Snellen chart) was performed. Afterwards, a short training phase followed before the rating of the stimuli started using the ACR scheme. In the training phase, possible image contents with typical distortions and the rating software were introduced to the participant. As rating software AVRateNG [3] was used. Some small modifications of the software were required to enable the applicability to images, e.g. the image was shown using a command-line video player (mpv^16^). Each image was presented using this software for 3 seconds and then rated by the participant according to the shown quality using the typical 5-point ACR scale. The overall subjective quality test lasted around 30 min. In total 21 participants took part in the study, mainly consisting of students and employees of the university.

After conducting the subjective image quality test, for each stimulus ratings in the range of [1, 5] are collected for each participant individually. In the following, all of the collected ratings are analysed and a comparison with objective image quality metrics is carried out.

#### Analysis of the ratings

To investigate the reliability of the laboratory test results, a simple outlier detection was performed. This outlier detection method uses a Pearson correlation threshold to identify outliers, which is widely used in state-of-the-art, e.g. in [40, 44, 81]. The used threshold was 0.8 (in other tests a value of 0.75 was used [86, 95]). The procedure is that based on all ratings the Pearson correlation of each individual rater is calculated and in case this correlation value is below the defined threshold this rater is classified as an outlier. For the lab test, no outliers have been identified, which is not uncommon in such a controlled lab test, because environmental and other influences are quite low.

**Fig. 7:**
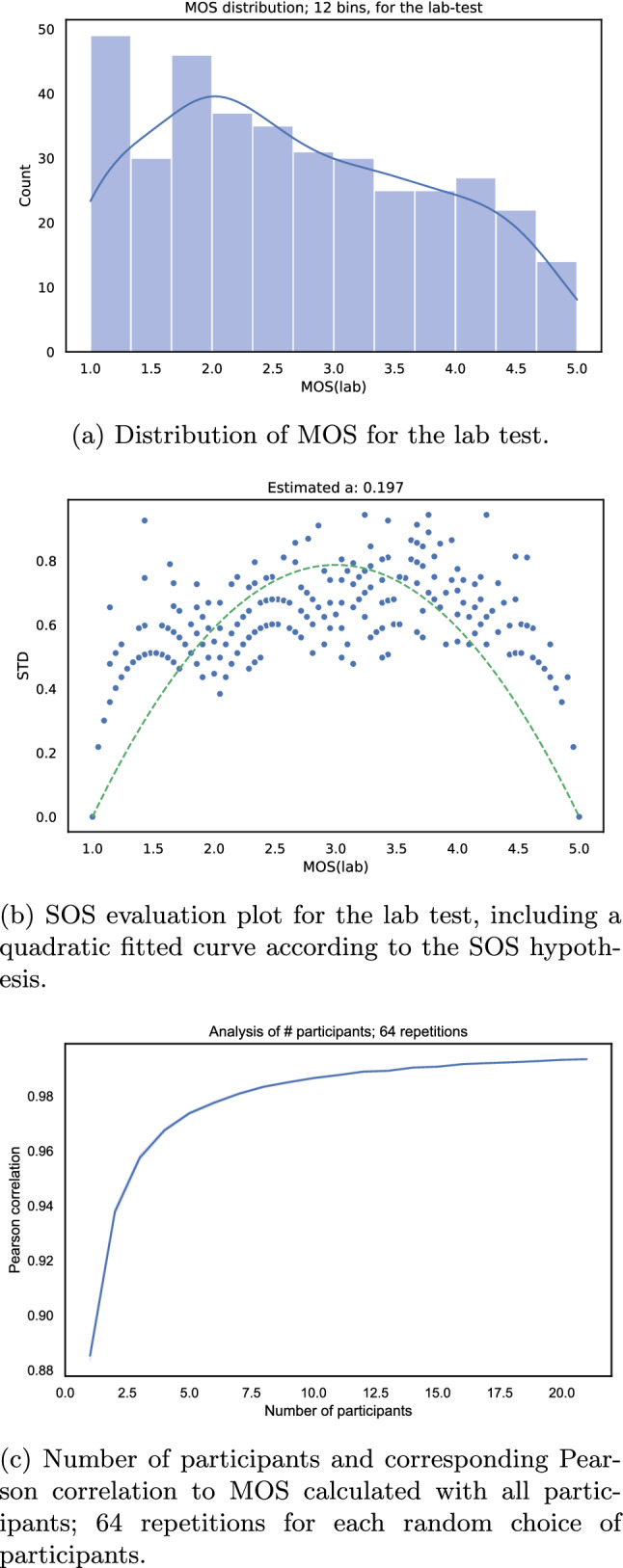
Evaluation of the lab test for image quality

For the overall score distribution, presented in Fig. 7a, a tendency toward uniform or lower-rated stimuli is recognizable. Here, it is required to consider the uniform sampling of VMAF scores in the filtering process as described in “Data sampling” section, it seems that lower VMAF scores are rated more critically by the participants. Another method to check the reliability of subjective tests is the SOS-analysis proposed by Hoßfeld et al. [34]. The general idea is to perform a quadratic curve-fitting on MOS and standard derivation values of the ratings, using the equation $SOS(x) = -a \cdot (x-5) \cdot (x-1)$. Afterwards, the scale factor of the quadratic function *a* refers to the reliability of the test results. For the conducted laboratory test, the estimated *a* parameter is $a\approx 0.197$, furthermore the corresponding SOS-plot is shown in Fig. 7b. The calculated value *a* is typical and valid for an image quality test, e.g., comparing to other reported values such as for the IRCCyN/IVC image test [34] where the value is $a\approx 0.17$. Furthermore other studies report a lower *a* value, however in these cases lower resolution images and other test methods, such as double stimulus approaches, are considered. For this reason, it can be concluded that the conducted subjective test has reliable results according to the SOS hypothesis. In addition to the SOS analysis, we also verified how many participants are required for the image quality test in the lab setting. Figure 7c shows the number of participants and the corresponding Pearson correlation, for each data-point 64 repetitions have been performed with a random selection of the participants. For example, it is visible, that with approximately 3 users per image, a Pearson correlation of more than 0.94 is reached, which indicates that not so many participants are required to reach a similar correlation as compared when conducting the same test in a similar lab setting, as reported by Pinson et al. [78].

#### Correlations with objective image quality metrics

Based on the conducted subjective test, it is possible to evaluate the performance of objective image or video quality metrics, i.e., VMAF [61, 69], ADM2 [57], VIF [101], PSNR, SSIM [112], and MS-SSIM [113]. For all images, the aforementioned quality metrics are calculated using the publicly available VMAF tool [69].

**Table 1:** Correlation values of several objective quality metrics to the subjective scores; values are rounded to 3 decimal places

Metric	Pearson	Kendall	Spearman
VMAF	0.919	0.757	0.925
ADM2	0.868	0.722	0.901
VIF scale2	0.861	0.740	0.911
VIF scale3	0.852	0.786	0.941
VIF scale1	0.846	0.674	0.859
MS-SSIM	0.701	0.658	0.851
PSNR	0.698	0.524	0.719
SSIM	0.658	0.802	0.948
VIF scale0	0.619	0.472	0.643

In Table 1 for all considered objective metrics, correlation values are presented, namely the Pearson correlation coefficient (pearson), the Kendall rank correlation coefficient (kendall), and Spearman’s rank correlation coefficient (spearman). The best-performing metric in this comparison is VMAF, directly followed by ADM2. However, ADM2 is used by VMAF as one of the underlying metrics. It was already analysed in [86] that ADM2 seems to have the strongest impact on the overall VMAF prediction for videos, thus a similar conclusion holds for image quality. In general, a high relationship between VMAF and the collected subjective scores is visible considering all three correlations.

Overall, the shown results are good, in comparison with for example the Pearson correlation for the same quality test that is conducted in several labs. As it is shown by Pinson et al. [78], the Pearson values are ranging from 0.902 to 0.935 for such inter-lab correlations. Based on this it can be argued that the VMAF prediction is within the expected error range. Thus it can be concluded that VMAF can be used for image quality prediction.

### Online test for image quality

Traditional lab tests are a well-established method to analyse the quality perception of participants. However, within the last years, crowdsourcing-based or online tests have increased in popularity [18, 35–37, 39]. Especially due to the fact that people with wider demographic backgrounds and more realistic viewing conditions can be recruited faster and at lower test costs, ensuring the overall sample of participants is more realistic. For this reason, the sampled images $ICF_{test}$ are additionally used in an online test.

#### Approach and challenges

In general, crowdsourcing-based or online tests introduce different aspects to the test design, conduction, and final analysis of the results [36]. Such differences originate from the diversity of possible crowd-users taking part in such a study, e.g., different end-devices, less constant environmental conditions, lighting conditions, distractions during the test participation, and even more [36]. Especially because of the variety of end devices, that are used to show the stimuli, it is not always possible to assume that participants own a 4K screen or are even using it for such a crowd test. Usual crowdsourcing participants have more common or even older hardware, that is not required to be up to the latest technology trends. However, the focus of the introduced sampled images and processing pipeline is still high-resolution image quality assessment, which would require a 4K capable screen. Clearly, some crowd platforms allow filtering users based on equipment, however, this would also influence the test results. To tackle this problem and further not exclude the majority of possible test participants, the sampled images of the dataset $ICF_{test}$ are pre-processed further. The main idea is to convert each 4K square stimulus into 4 patches with a square size of 1080*p* each. In addition to solving the presentation dilemma, such an approach will also enable the possibility to analyse the connection between patch-based and overall image quality considering patches with higher-resolution, in contrast to [114], where only lower resolution images are used.

As test software, similar to the lab test, a modified AVRateNG [3] version was used. These modifications resulted in AVRate Voyager [24], see “Remote testing framework” section. In addition to the ratings for each stimulus, further demographic information, the used browser, and browser size are stored for later analysis of the remote participants.

Moreover, the original sampled 371 images of the $ICF_{test}$ set resulted in 1484 Full-HD sized patches. The lab test was designed to last around 30 min for the complete rating of all images, whereas rating all of the 1484 patches is neither suitable for a lab test nor for a crowdsourcing or online test. Here, another modification to traditionally conducted full-factorial lab tests is required. In the online test each participant rates 150 uniform random sampled Full-HD patches, referred to as part-factorial design. Pre-tests showed that approximately 10–15 min are required to perform the designed remote test, which is necessary as the overall duration has an influence [36] on the rating quality. Moreover, an explicit training phase was removed, to shrink the overall time for the test even more. This modification also results in the need for more participants in the online test, so that each shown patch is rated by around 20 participants in the ideal case. To rate all included images of the lab test it is thus required to have approximately 200 participants taking part in the crowd test, following the described part-factorial design.

In total 238 subjects took part in the study to rate image quality, they were recruited within the university, to also ensure comparability with the conducted lab test.

In the following, only participants who finally rated images are considered to be valid participants, all other participants were already removed (e.g. participants who just filled out the first form and never rated an image). First, the participants themselves are investigated in more detail, this is required for the design of future crowdsourcing-based tests.

#### Analysis of the crowd users for the image quality test

**Fig. 8:**
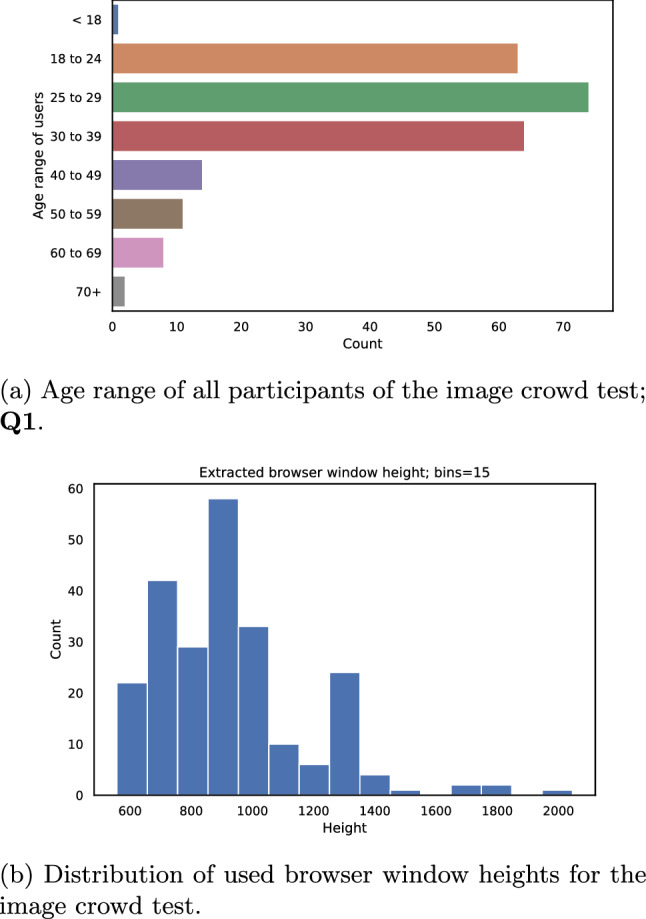
Evaluation of the users of the image crowd test

**Fig. 9:**
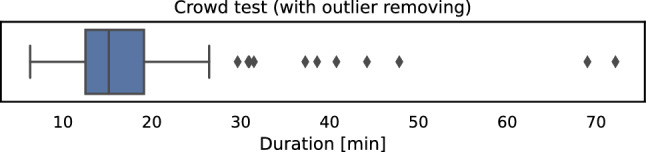
Duration required for the image crowd test; most participants needed $\approx$15 minutes for the test

The participants have been asked to fill out a demographic form at the beginning of the test, the rationale of this questionnaire was to pre-cache the images during the time it takes to answer the questions. In total the following four questions (**Q1**, **Q2**, **Q3**, and **Q4**) have been asked.

- **Q1**: “What is your age?” (8 answer categories possible),
- **Q2**: “How good is your vision?” (6 answers possible),
- **Q3**: “Which option best describes your environment?” (3 possible answers),
- **Q4**: “What type of device are you now using?” (4 categories)

In Fig. 8a the results for the age question (**Q1**) are shown, it is visible that the recruited participants form a “younger” crowd, whereas even some older participants took part in the study. The next question (**Q2**) was a self-report about vision. The majority of the crowd users either selected excellent or good vision, whereas some selected worse options. **Q3** refers to the environment of the participants, here also a self-report was used, as other approaches were considered too intrusive regarding test subjects’ privacy for this test. Most participants were either in a quiet room or stated to be just minimally influenced by noise. The last question (**Q4**) refers to the user’s device, it was strongly recommended to use a PC or Notebook for the test. In addition, the rating software used a check of the browser window size to ensure a minimum height and width, this check enforced that it is not possible to run the test on a smartphone or tablet respectively. This decision was made to include only participants with larger screens because in a pre-test it was observed that some participants may use devices with very low display resolution.

In addition to the questionnaire, AVrate Voyager also collected some generic information about the crowd participant. Here, only the window size and the used browser agent have been stored. In Fig. 8b the used window heights are shown. There are a few participants with a 4K screen within the crowd. Most of the crowd users used a window height of approximately 720–1000 pixels, which leads to an HD or Full-HD native display resolution. So the general assumption in the preparation of the crowd test, to only handle 1080*p* patches, is mostly confirmed. In addition to the gathered answers in the questionnaire, the overall duration of the crowd test can be estimated, as shown in Fig. 9. Most of the participants needed about 15 minutes to conduct the test which was approximately the time that was initially planned for the crowd test.

#### Ratings and score distributions

**Fig. 10:**
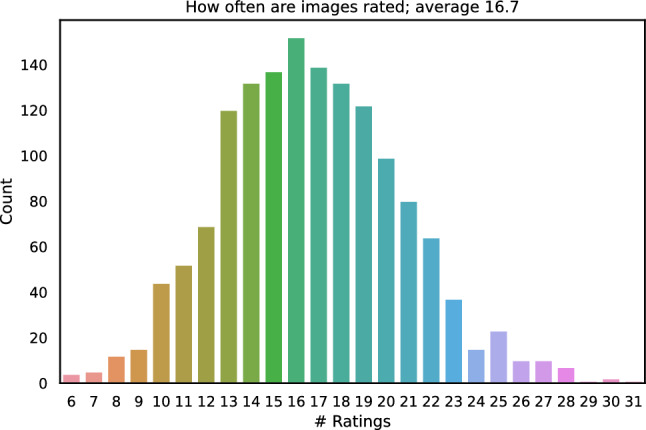
Count distribution of how often images patches were rated

The crowd test provides the participants with 150 out of 1484 randomly selected to-be-rated image patches. Figure 10 shows how often image patches are rated. On average each image patch is rated by $\approx 17$ participants. In total 1439 patches were rated at least 10 times. 45 image patches were rated less than 10 times.

**Fig. 11:**
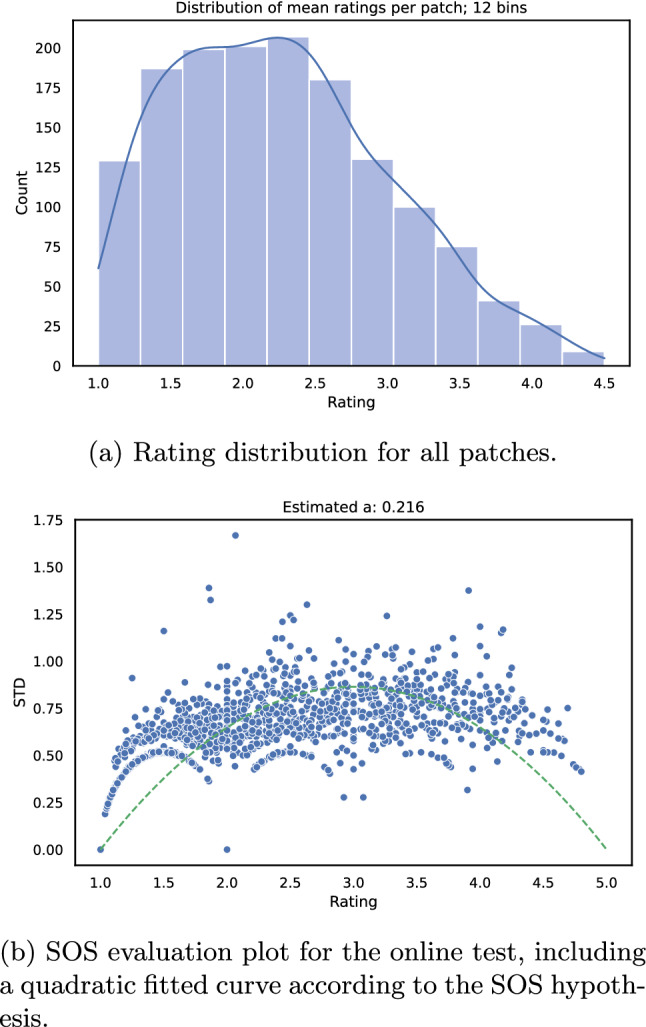
Evaluation of the image online test

Furthermore, in Fig. 11a the distribution of MOS for all patches is shown. The rating distribution is similar to the laboratory test (see Fig. 7a). However, there are fewer cases where a high-quality rating was selected by the participants. The reason for this is that some patches are difficult to rate due to compression artefacts, or because the patches are hard to recognize (e.g. a black patch). Also, this could be a result of the patching approach, because it decomposes the “global” picture and participants are less able to understand the image itself.

Similar to the lab test, an SOS analysis [34] was carried out, compare Fig. 11b, where the mean and standard deviation values are shown for all patches. An *a* value of $\approx 0.22$ was estimated, which is similar to web surfing or video streaming tests [34]. Furthermore, a shift to lower ratings is visible, similar to the distribution plots 11a. This can be explained by, for example, the more critical view of the individual participants.

#### Correlations with lab test

It is further important to consider that each original image is split into four patches, which implies that for one image four individual ratings are collected within the crowd test. To compare the conducted crowd and lab tests, first, each patch rating is compared to the lab test ratings, and later a mean rating of all patches.

**Fig. 12:**
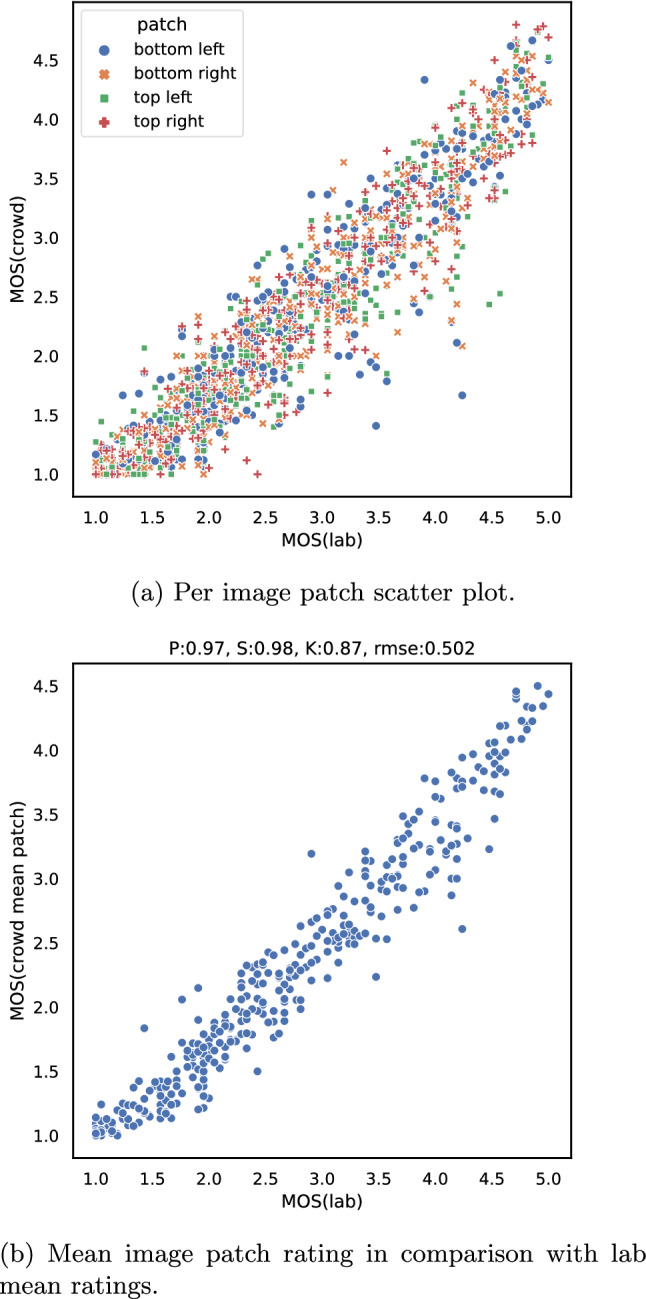
Comparison of lab and online test for image quality

In Table 2 correlation values, Pearson, Kendall and Spearman, along with *rmse* values for each patch compared to the lab tests results are presented. First of all, the individual patch mean rating correlates high with the lab test ratings, compare also Fig. 12a. However, the performance of all individual patches is nearly identical, thus it can be concluded that individual patches can be used individually for image quality evaluation. This is similar to results of Göring et al. [21], where e.g. centre cropped video frames showed similar results for the overall quality estimation in the case of videos. As next, the mean rating of all patches per image is considered to form the overall mean quality rating per full image. In Fig. 12b the corresponding scatter plot is shown. The combination of all patch ratings leads to an overall better correlation (Pearson value of 0.97) than individual patches and an overall lower error (*rmse* of 0.502). However, in general, a tendency for lower ratings for image patches can be observed, because the overall rating range in the case of the crowd test is [1.0, 4.5] in contrast to [1.0, 5.0] for the lab test. Here, it should be noted that in usual model development a linear fitting would be performed, to normalize the two tests. Such a linear fitting is already captured within the Pearson correlation values. Moreover, the high correlation of mean patch ratings compared to the lab test also indicates that participants seem to not focus on individual image aspects for their quality rating.

**Table 2:** Correlation values of individual image patches in comparison with lab test ratings, furthermore *rmse* is calculated

Patch	Pearson	Spearman	Kendall	RMSE
mean	0.970	0.980	0.870	0.502
Top right	0.954	0.954	0.823	0.527
Bottom right	0.946	0.950	0.811	0.551
Top left	0.941	0.948	0.805	0.565
Bottom left	0.933	0.934	0.794	0.567

### Observations

To sum up, it was shown that lab and online tests can be used for the image quality assessment problem. In the case of the online test, it was required to use patches instead of full images so that participants are able to display the images properly on their lower-resolution screens and to reduce the overall number of to-be-rated stimuli. It was found that the majority of participants own a 720*p*-1080*p* screen, which should be considered in future online tests. In contrast to the lab test, which had a duration of 30–40 min, the online test was shorter (about 10–15 min) and this necessitated including more participants in the online test. Furthermore, it was shown that VMAF can be used to objectively assess image quality.

## Short-term high resolution video quality assessment

Following the image quality assessment study, short-term video quality assessment of high-resolution videos is considered as a second use case to be tested in an online setup. In general, obtaining valid quality ratings for high-resolution video quality poses several problems. Example issues are that streaming of such high-bandwidth content may not be feasible for some users, or that remote participants do not have an appropriate and high-resolution display device. This section describes the dataset and platform used as well as the required pre-processing of the encoded videos to conduct a short-term video quality assessment test with the online paradigm. We used AVrate Voyager to implement the online test. Furthermore, we compare the results with a previously conducted and published dataset of a short-term video quality lab test, which formed the basis of the online test.

### Short-term video dataset

**Fig. 13:**
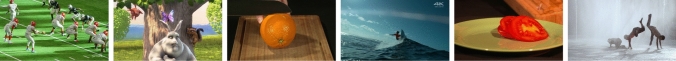
Overview of the source videos used for the video quality evaluation

For the online test, we used the videos from test_1 of the AVT-VQDB-UHD-1 [86] dataset. Accordingly, six different source videos of a duration of 10 s each were used. In Fig. 13 an overview of the used videos as thumbnails is shown. The source videos have a resolution of 3840 $\times$ 2160 pixels and a framerate of 60 fps. They were encoded with three different codecs, namely, H.264, H.265, and VP9. For each of the codecs, multiple (bitrate, resolution) conditions were used to encode the videos, resulting in a total of 180 processed video sequences (PVS). The framerate of the encoded videos was kept at the source sequence framerate of 60 fps. In the original lab test, a total of 29 participants took part. According to [86], there were no outliers, based on the criterion of 0.75 Pearson correlation between individual subjects and the overall ratings. More details of the lab test are described in [86].

### Video pre-processing

The encoded video segments were decoded as described in the publicly available implementation of AVT-VQDB-UHD-1 [86]. This decoding involves a lossless up-scaling of the encoded videos to the source sequence resolution and framerate, which is referred to as the AVPVS in the following. In a typical lab test, hardware capable of seamlessly playing out the AVPVS can be ensured. Whereas, in a crowdsourcing or online context, neither appropriate playout hardware nor a UHD-1/4K capable display device can be guaranteed. Since a variety of screen sizes may be used across the participants in an online test, the fixed UHD-1/4K screen and target resolution used in the AVT-VQDB-UHD-1 tests by [86] will exceed the available resources in many cases, as it also has been verified in the conducted online image quality test.

As a consequence, we chose to display a 540*p* centre crop of the AVPVS which is $\frac{1}{16}$th the number of pixels of the AVPVS in the test. This is based on the results by Bosse et al. [8], who concluded that a 128 $\times$ 128 pixels patch out of a 512 $\times$ 512 pixels image is sufficient for subjective image quality assessment and the observations by Göring et al. [21] on different pre-defined centre crops for full reference model evaluation. However, there still exists the issue of playing out the 540*p* centre-cropped AVPVS in the browser. To reduce the data rate of the AVPVS and thus ensure a seamless playout in the browser, we selected to encode the 540*p* centre cropped version using H.264 with a CRF of 22 with a chroma-subsampling of 4:2:0 and 8 bit. A CRF of 22 guarantees both the smooth playout in the browser while entailing negligible loss in the visual quality of the AVPVS. For example, a UHD-1/4K video (e.g. big bucks bunny $VMAF=94$ or cutting orange $VMAF=93$ of the AVT-VQDB-UHD-1) encoded with CRF 22 results in a VMAF score of $>93$ which is supposed to be high quality. In the context of the *P.NATS Phase 2* competition [81], also a CRF encoding was used for the playout of stimuli in the case of mobile devices.

### Test procedure

The online test was designed with the intention of restricting the total duration of the test to below 15 min. At the beginning of the test, each participant was asked to fill out a form consisting of information regarding the age range, self-judged visual acuity on an ACR-scale, the device type being used in the test, and also about the environment the participant is in when doing the test. We chose to ask only a minimal number of questions to limit annoyance, and all data is stored in an anonymized manner to ensure data protection using the AVrate Voyager framework. The mentioned questionnaire is similar to the one used in the image quality test.

In the online video test, it is also not possible to ask the participant to rate all stimuli. Therefore, we use the part-factorial approach of asking each participant to rate 30 randomly selected PVSs out of the overall number of 180. These 30 PVSs were pre-loaded while the participants answered the pre-test questionnaire. There was no training phase to keep the test duration within 15 min.

### Video online test

In the following, we briefly analyse the pre-questionnaire and other passively collected data of our short-term video online study.

**Fig. 14:**
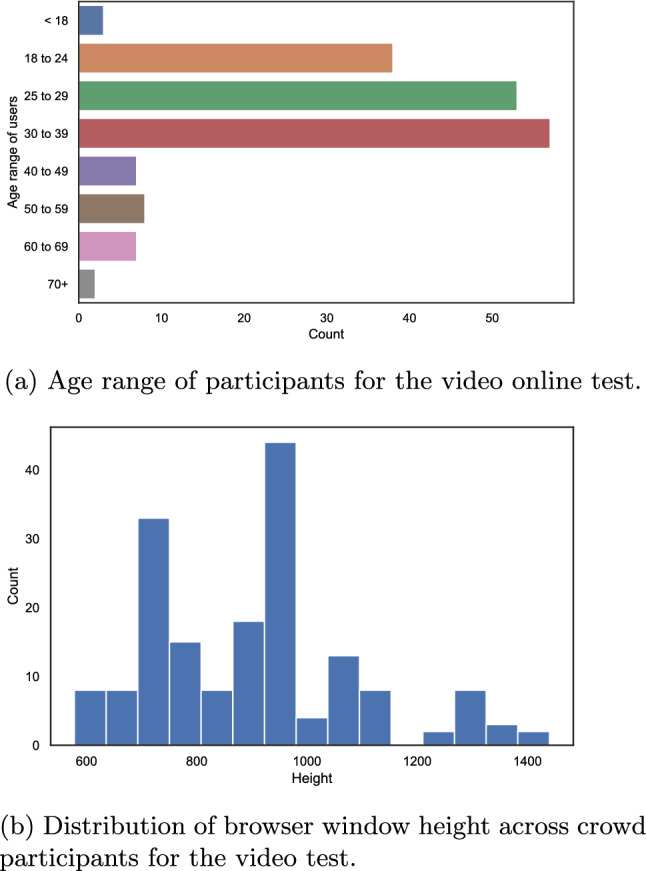
Evaluation of the users of the video crowd test

Most of the participants self-reported a good vision and that they took part in the test in an environment with “less distractions”. In addition, the participants mainly carried out the video quality assessment test either on a laptop or on a desktop PC. Other devices may not pass the height and width check of AVrate Voyager, and it was recommended to use a PC or laptop for the test. The majority of the participants were in the age range from $18 - 39$ years, compare Fig. 14a.

While the participant filled in the questions, we pre-cached the videos and collected the dimensions of the used browser window. The distribution of the extracted height of the window in which the video was viewed is shown in Fig. 14b.

It can be seen that most of the subjects used the recommended screen resolution of 720*p* to watch and rate the videos. Moreover, the results are similar to the image quality online test, compare to “Analysis of the crowd users for the image quality test” section. An interesting observation is that there are very few subjects, $\approx$18%, who used a device with a resolution of Full-HD or higher. This indicates that running an online study for quality assessment of higher-resolution videos is challenging. The device distribution substantiates the need for a test method such as the patch-based approach used in our online test.

**Fig. 15:**
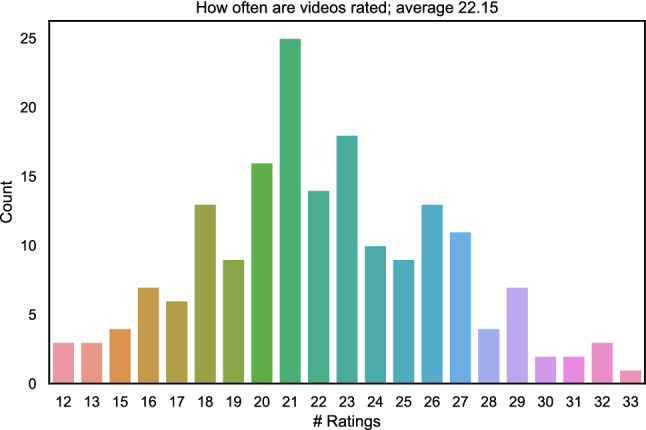
Count distribution of how often PVSs were rated; e.g. x=24 and y=10 means that 10 PVSs were rated 24 times in the crowd test

A total of 175 subjects participated in the online study. The participants in this study consisted of people recruited from the university body via email reflectors (reaches students and staff). To determine the outliers in the test, we used the criteria of Pearson correlation coefficient (PCC). In the case of a PCC lower than 0.75 of the individual subject’s ratings to the mean ratings across all subjects, that subject was considered as an outlier. Based on a threshold of $PCC = 0.75$, 19 outliers were detected and the ratings from these participants were removed for further analysis. A total of 3987 ratings were obtained after outlier removal, with an average of 22.15 ratings per PVS. We analysed how often each PVS is rated and created a histogram of these counts that is shown in Fig. 15.

Furthermore, since each participant rated only 30 randomly selected PVSs out of the 180, further analysis was performed to determine the minimum number of subjects needed to have each PVS rated at least once. For this purpose, we performed the analysis of the test results with 64 different randomizations of the order of participants’ ratings and averaged the results, indicating that for the given test, it took 39 participants to have each PVS rated at least once, and 144 participants to have each PVS rated at least ten times.

### Lab versus online test comparison for video quality

**Fig. 16:**
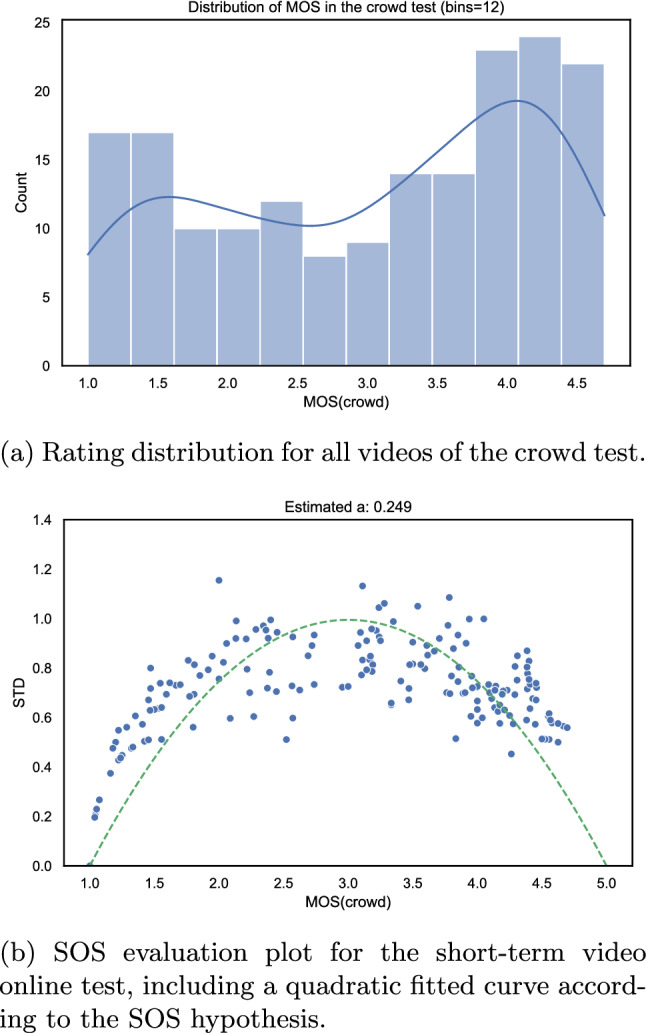
Evaluation of the short-term video online test

**Fig. 17:**
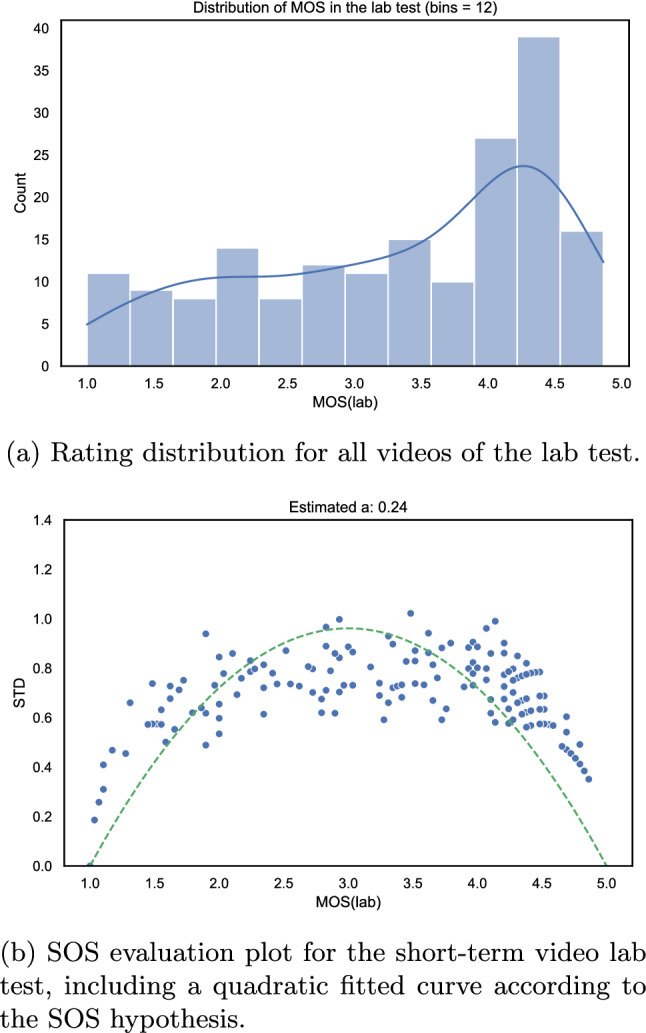
Evaluation of the short-term video lab test

In the following part, we will present the comparison of the results of the short-term video online and the lab tests.

The distribution of the MOS values of both lab [86] and online tests are shown in Fig. 16a and Fig. 17a respectively. From the somewhat more negative ratings in the case of the online test, it can be concluded that remote participants are more critical as compared to the participants in the lab test while rating the videos. This can likely be attributed to the fact that in the online test, the 540*p* centre-cropped versions of the video were rated by the participants and not the full UHD-1 version like it was the case in the lab test. As a consequence, the participants in the online test focused on a smaller area of the video and hence may have been more sensitive to any kind of video distortions. Further, since only a small portion of the video was shown, semantic information and a full understanding of the sequences were not enabled. Therefore, test subjects may have had a stronger focus on the video-signal quality than with the full video frame being shown.

We further performed an analysis of the distribution of standard deviations over the MOS (SOS analysis [34]) to estimate the similarity between the lab and online tests. The SOS-plots are shown in Figs. 16b and 17b for the online and lab test respectively. For the lab test, an SOS parameter $a_{lab}=0.240$ was estimated, and for the online test of $a_{crowd}=0.249$. Both values are within the same order of magnitude and hence a strong similarity between both tests can be concluded.

**Fig. 18:**
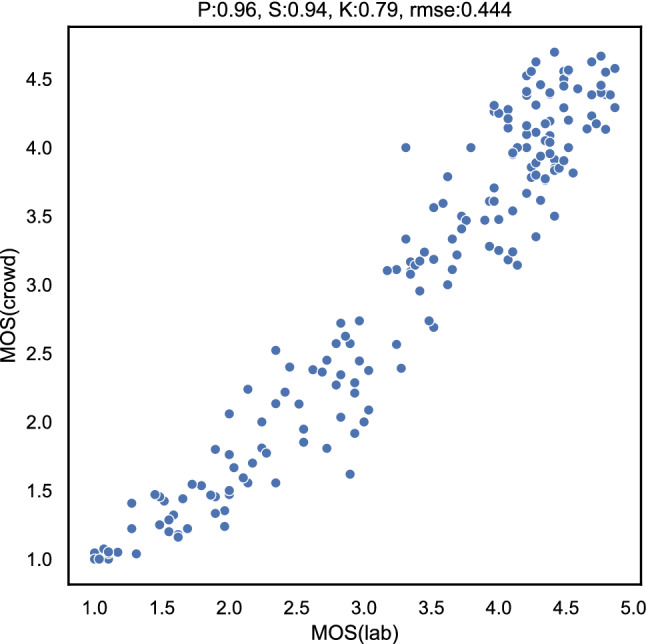
Scatter plot of the MOS values from lab [86] and online short-term video quality tests (mean_crowd)

Figure 18 shows the comparison of the mean opinion scores (MOS) from the lab and online tests. It can be seen that there is a very high correlation between the two tests, with a Pearson correlation of 0.96, which is comparable to the performance reported for cross-lab testing for video quality assessment [78]. This indicates the validity and reliability of our online approach and the modifications of using a 540*p* centre cropped version of a UHD-1 up scaled video to evaluate the video quality.

**Table 3:** Per-source comparison of lab [86] and online short-term video test results

Source	Pearson	Spearman	Kendall	RMSE
american_football	0.98	0.96	0.85	0.535
bigbuck_bunny	0.99	0.95	0.84	0.305
cutting_orange	0.96	0.89	0.75	0.276
surfing_sony	0.97	0.96	0.86	0.689
Vegetables	0.96	0.88	0.73	0.346
water_netflix	0.99	0.98	0.89	0.444

Furthermore, we compared the performance of the two test paradigms on a per-source basis. As can be seen from Table 3, also at a per-source level there is a very high correlation between the two tests. All videos individually have a Pearson correlation of at least 0.97.

**Fig. 19:**
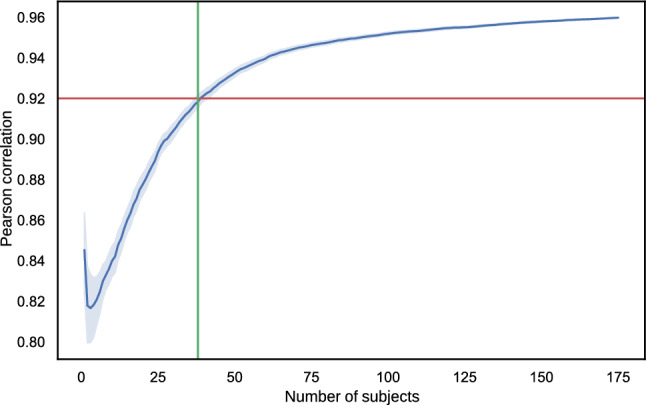
Correlation between lab and online tests as a function of the number of participants in the video online test

In addition, we analysed the correlation between the lab and online tests as a function of the number of participants in the online test, compare Fig. 19. The reason for this is to evaluate how many participants are required in such a non-full-factorial online test. For this analysis, we randomized the order of the participants of the online test and calculated for the first *n*-th participants ($0<n\le 156$) the Pearson correlation to the MOS of the lab test. We repeated this randomization 64 times and calculated mean values and the corresponding confidence intervals, as shown in Fig. 19. It was found that for a correlation between the tests greater than 0.92, a minimum of 39 participants is required, then leading to a similar correlation as found for cross-lab test comparisons [78]. It should be noted that it took 39 participants to rate each PVS at least once as described earlier.

### Observations and findings

To enable reliable crowdsourcing or online studies for quality assessment of high-resolution videos, we propose a patch-based test method using the centre cropped version of the full UHD-1 video, with a crop height of 540 pixels. We used videos from test_1 of the publicly available AVT-VQDB-UHD-1 [86] for this purpose. The results of the crowdsourcing test and the comparison with the corresponding lab test indicate high similarity between both tests, with an inter-test correlation of the MOS of 0.96. Moreover, the SOS analysis resulted in both cases with similar values. So we were able to verify the reliability of the online test. The two main modifications for the online tests can be summarized as using the centre-cropped version of the videos and reducing the overall number of stimuli rated by one participant to 30 instead of 180. The evaluation shows that both changes result in similar results as it would have been the case for a traditionally conducted lab test. Similarly to the image test, results showed, that participants which are recruited online may have only lower resolution screens (720*p* to 1080*p*), which is important for the design of such remote tests.

## Long-term audiovisual quality assessment

As with the short-term video quality assessment studies, overall HAS session quality assessment studies can be conducted in a non-lab setting. However, this comes with its own set of challenges. One major challenge in conducting such tests with videos of longer duration is the number of PVSs that each participant in a non-lab setting is asked to rate. Unlike short-term video quality assessment where it is still possible to sub-sample the PVSs to ensure that each test subject views and rates videos covering the overall quality range, it becomes more difficult when using videos of longer duration as the overall number of PVSs that a participant rates is limited. Hence, it is needed to compare the subjective ratings between lab- and crowd-based tests to investigate the rating behaviour of the subjects in these two different scenarios and thereby check the reliability of the crowd-based studies in comparison with lab tests.

In this section, a test for overall quality assessment of a HAS session conducted in a crowdsourcing environment following the approach described in “Test procedure” section is presented.

**Fig. 20:**
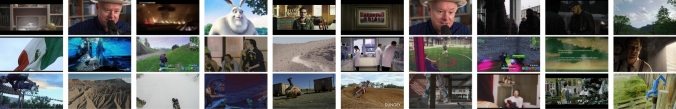
Overview of the source videos used for the long-term audiovisual quality evaluation

### Long-term video lab test

**Fig. 21:**
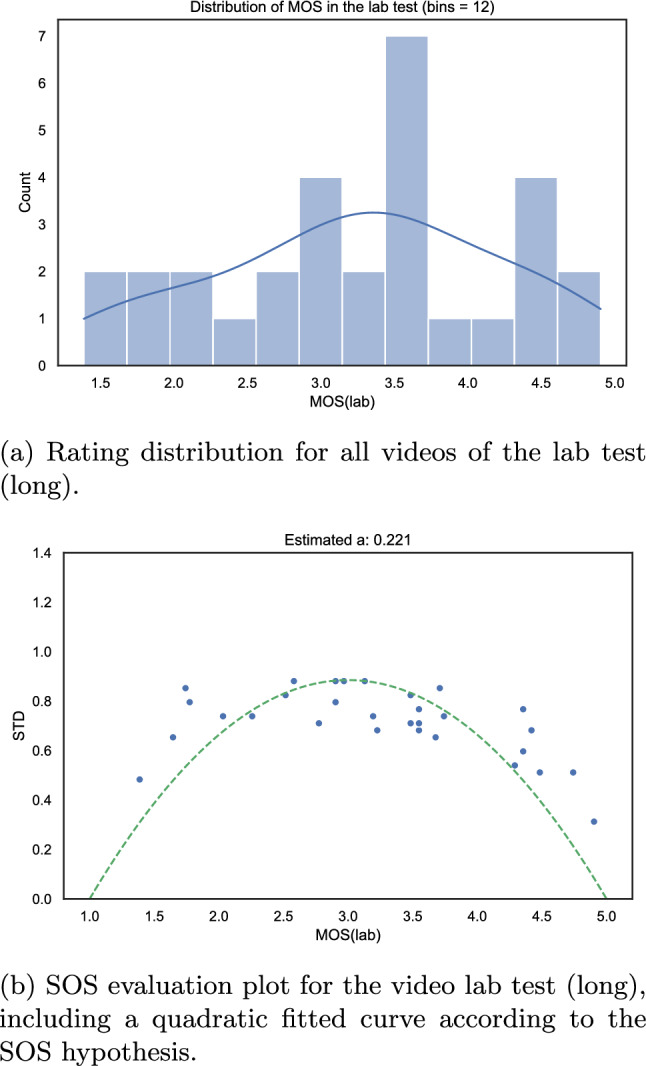
Evaluation of the video lab test

The original lab test was designed and conducted as part of the *P.NATS Phase 2* competition that resulted in the ITU-T P.1204 series of Recommendations [45, 81]. The test focused on assessing the overall quality of a 2 min videos that were distorted with degradations related to coding and stalling-related events. Furthermore, the test follows a design based on the “immersive” paradigm [76] in which the participants newer view the same source stimulus more than once. Hence, 30 different sources of 2 min duration were used and an overview of the covered source videos is shown in Fig. 20. All the sources had a resolution of 3840 $\times$ 2160. The encoding conditions were defined by varying the parameters related to the encoding of the video segments, initial stalling duration, number and duration of stalling events, and number of quality switches to create the PVSs. Each subject was asked to rate a total of 30 PVSs. All the PVSs were up-scaled to the native UHD-1/4K resolution of 3840 $\times$ 2160 and converted to a lossless video codec. The PVSs were displayed on a LG OLED55C7D 55" screen. A total of 31 participants took part in the study. An outlier detection was performed with the criterion of $PCC = 0.7$ and no outliers were identified.

The overall MOS distribution and the SOS analysis of the lab test are shown in Fig. 21.

### Long-term video crowd test

In addition to the lab test, we also performed an online test for long-term video quality assessment. The study was conducted using the Clickworker^17^ crowdsourcing platform. The countries from which the participants were recruited were restricted to Europe. For the rating task, AVrateVoyager [24] was used. All the checks mentioned in “Test procedure” section for the short-term video quality crowd tests were also repeated in this test.

#### Pre-processing of long-term videos

The encoded videos were decoded as done for the short-video segments along with lossless up-scaling of the encoded videos to SRC resolution and framerate. Furthermore, a 720*p* centre-crop of the video was extracted to be played out in the rating task. The decision for using a larger centre-crop as compared to the short-term video quality online test was to provide more context in terms of the content as the duration of the video was longer. This centre-crop version of the video was then encoded with a CRF of 22 using H.264 with a yuv420 8-bit pixel format to ensure a playout with a web browser. Also, the decision of using the centre-crop and the chosen CRF-based encoding was primarily motivated by the challenges outlined in “Test procedure” section.

#### Test procedure

As with the short-term video quality test, the overall test duration was restricted to 15 min. This included the time required to fill in the pre-test questionnaire which consisted of the same questions asked in the short-term online study. Unlike the short-term video test, this test had a training phase consisting of one training video with all the possible degradations related to a typical HAS session. Such degradations were initial loading delay, quality switches, and stalling events. The motivation for including the training video was to familiarize the test participants with these degradations while evaluating the video quality. Furthermore, the subjects were provided instructions explicitly to consider only the degradations and not the content to evaluate the overall quality of a session. There were no degradations introduced to the audio. A total of 100 crowd workers were recruited via the Clickworker platform and as a pragmatic approach, each crowd worker was asked to rate 5 PVSs that were randomly selected out of the overall number of 30 PVSs. These 5 PVSs were pre-loaded while the subjects answered the pre-test questionnaire, to avoid further loading delay for each PVS later.

**Fig. 22:**
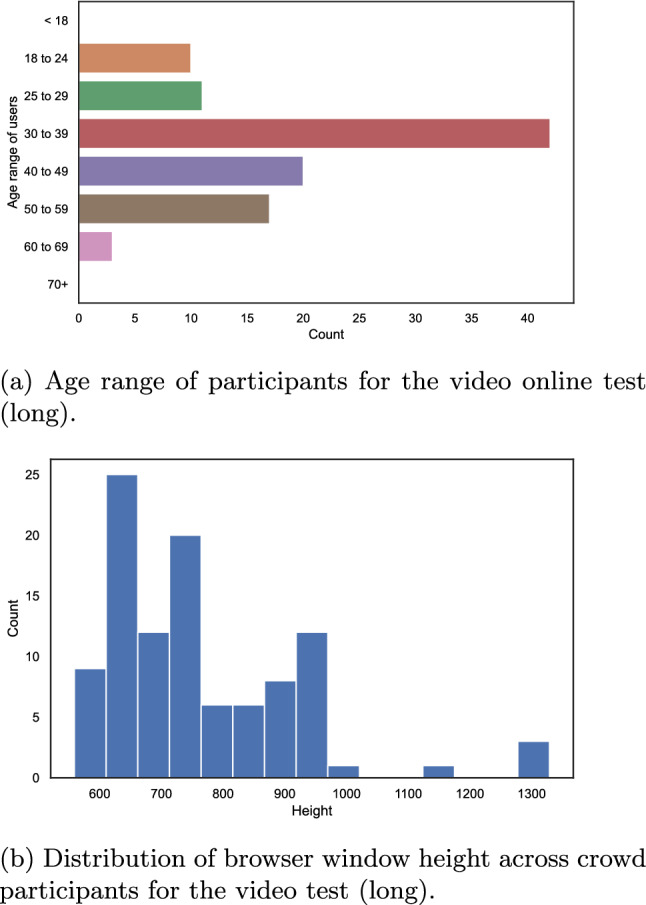
Evaluation of the users of the video crowd test (long)

**Fig. 23:**
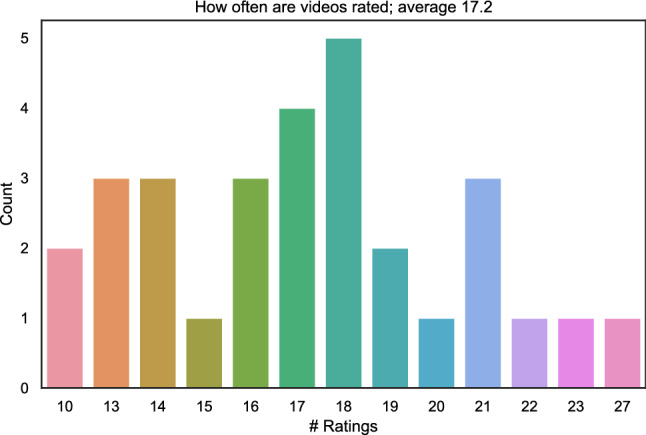
Count distribution of how often PVSs were rated; e.g. x=18 and y=5 means that 5 PVSs were rated 18 times in the crowd test (long)

**Fig. 24:**
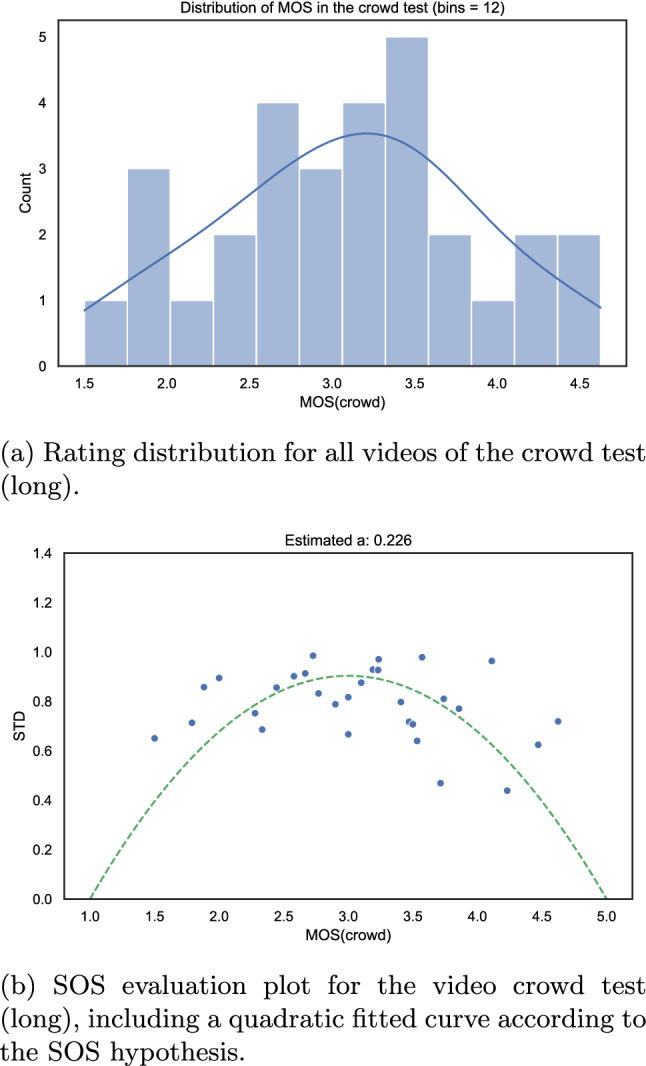
Evaluation of the video crowd test

### Results and evaluation

The results are presented in two parts. The first part consists of the results of the crowdsourcing study while the second part deals with comparing the results of the lab and crowdsourcing tests. The focus of the comparison is to demonstrate the applicability and reliability of extending the centre-crop-based video quality assessment for long-duration videos with HAS-related impairments.

#### Crowdsourcing test analysis

In contrast to the short-term video test, the subjects were recruited from Clickworkers and thus paid. Most of the participants did the test alone in a quiet room with a significant proportion of them doing it on their laptop or desktop and self-reporting good to excellent visual acuity. It should be noted that visual acuity determination was based on self-reporting on a 5-point ACR scale. Also, there is a good distribution in the age range of participants taking part in the study, compare Fig. 22a.

In addition to gathering responses from the participants using the pre-test questionnaire, the dimensions of the used browser window were also collected in parallel to the subjects answering the questionnaire, see Fig. 22b. It can be observed that similar to the short-term video test, very few subjects ($<10\%$) used a screen with 1080*p* or higher resolution, thus justifying the decision to use a 720*p* centre crop for quality assessment.

In addition to this, an analysis of how often each PVS was rated was performed and is illustrated in Fig. 23 and the average number of ratings for each PVS was 17.2.

#### Comparison crowd and lab test for long-term video

A comparison of the lab and crowd tests is described in this section to show whether the centre-crop approach can be used for the assessment of long-duration videos with HAS-related impairments. Figures 21a and 24a shows the distribution of the MOS in the lab and crowd tests respectively. As with the short-term video test, the crowd participants are more critical than the lab subjects also most likely because they had a smaller region to focus on and hence would have been more critical to the video-related degradations. As with the short test, this hypothesis has to be further investigated in follow-up studies.

Furthermore, an SOS analysis [34] of both the lab and crowd tests was conducted to estimate the similarity between the two tests. As illustrated from Figs. 21b and 24b, it can be concluded that both tests have a strong similarity and the same order of magnitude of the SOS parameter with $a_{lab}=0.221$ and $a_{crowd}=0.226$ respectively, which indicates a strong similarity between the tests.

**Fig. 25:**
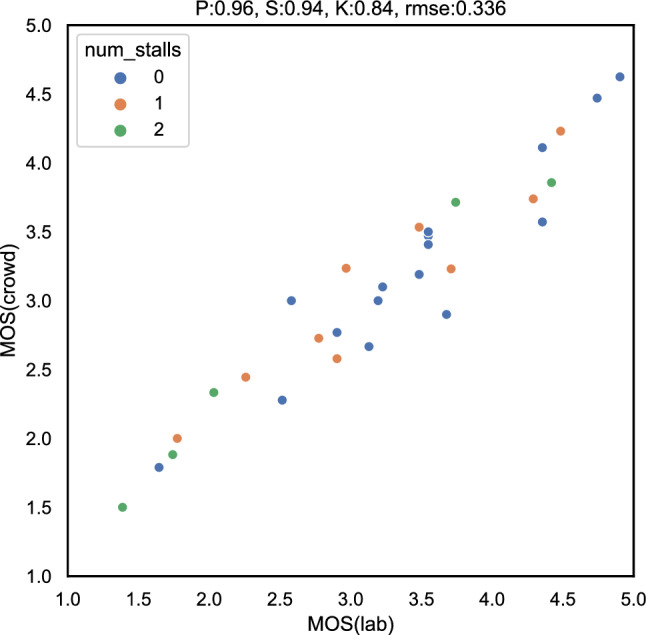
Scatter plot of the MOS values from lab and crowd video quality tests (long)

In addition to these analyses, a comparison of the MOS from the lab and crowd tests was performed and is visualized in Fig. 25. A high correlation can be observed between the two tests with a PCC of 0.96. Such a Pearson correlation value is similar to the repetition of the same test in a lab scenario, as reported by Pinson et al. [78]. This high correlation indicates the validity and reliability of extending the crowdsourcing approach to assess the overall quality of a HAS session.

Also, it can be observed from the high value of the Spearman correlation of 0.94 that the rank order of the PVS is similar in both of the tests, and the general agreement in assessing the cases related to stalling events further establishes that the instructions provided to the participants were sufficient.

### Observations

After using the proposed centre-crop-based approach for the task of short-term video quality assessment of high-resolution videos, the approach is extended to assess the overall quality of a HAS session. Unlike short-term video quality assessment, this use case requires videos of a longer duration, typically $\ge 1\,min$. Furthermore, participants are required to take into account the video and audio quality switches and stalling events in the quality assessment task. Although the participants are instructed to not take content into account in the quality judgement, sufficient care needs to be taken to not induce boredom while choosing to use the centre-crop-based approach in such a study. Hence, a 720*p* centre-crop is used in this test to give more context to the subjects for their quality rating. For this test, $2\,min$ videos from the corresponding lab test conducted as part of the *P.NATS Phase 2* competition are used. Also, in contrast to the short-term test where unpaid subjects from the university body were used, this test was conducted with paid participants recruited using the Clickworkers platform. Similar to the short-term video quality test, a comparison between the lab and crowd tests shows a high degree of similarity in terms of MOS with a PCC of 0.96. In addition to this, the SOS analysis also further confirms the agreement between the tests with the corresponding SOS parameter values.

## Discussion and conclusion

Quality assessment of higher-resolution content, such as videos or images, is usually performed via well-controlled lab tests. However, instead of lab tests, crowd, online or out-of-the-lab tests can also be used to collect annotations regarding visual quality and these out-of-the-lab tests have increased in popularity in the last years [39, 82, 105] to mitigate the drawbacks of lab tests in terms of time consumption and costs incurred. Within the context of higher-resolution multimedia content and out-of-the-lab testing, we analysed three different aspects of quality assessment, namely, image, short-term video, and overall HAS session quality. To perform this analysis, two different research questions were identified, one related to the test methodology and the other to the reliability of online tests. Also, for all three aspects, we performed a lab and a remote test and compare the results to address the question of the reliability of the out-of-the-lab tests.

In general, some challenges arise when transforming a lab test into an online test. For example, suitable remote testing software is required, for this reason, we developed our own framework namely AVrate Voyager, which is publicly available. Furthermore, such online tests should also be shorter in duration in contrast to lab tests, e.g., following the guidelines by Hossfeld et al. [36]. To reduce the test duration, in our described tests, the participants only rate a randomly selected subset of all test stimuli.

Another important aspect that needs to be considered is that typical remote participants may not have high-end devices to display the stimuli. This leads us to adapt conventional test methodologies to overcome these limitations. The adaptation that we use in our studies is using a patch-based approach for images and a centre-crop-based approach for videos. We evaluate and verify these adaptations with three different studies related to image, video, and overall HAS session quality assessment.

First, a study considering image quality was conducted. Here, a comparison of the remote and corresponding lab tests has been carried out. The UHD-1 images have been compressed using H.265, and image patches have been shown to 238 participants in the online study. The participants were recruited from the university body and were unpaid. From the results, it can be concluded that the online and lab tests are similar considering the results in terms of both Pearson correlation and the magnitude of the corresponding SOS [34] parameter. In addition to this, both tests also show, that VMAF is a suitable method to assess image quality. The image quality evaluation test is to be seen as the first iteration of the described online remote testing evaluation. Furthermore, analysis of the pre-test questionnaire shows that the used window size of all participants is limited. It could be observed that the majority of participants had lower resolution screens ranging from 720*p* to 1080*p*.

Following this, a short-term video quality assessment test was conducted in an online setting. For this, an approach based on a pre-defined centre crop is used. In particular, for this test, a 540*p* centre-crop of the original 2160*p* video was chosen to be displayed to the subjects. The videos used in the online test originated from the test_1 of our publicly available AVT-VQDB-UHD-1 [86] dataset. This was done to have a comparison between the lab and online tests. In total 175 subjects took part in the online tests and like in the image quality study, they were recruited from the university body and were unpaid. Similar to the image quality test, we compared the remote test with a previously conducted lab test for the case of short-term video quality evaluation. The results revealed that both test results were similar considering the Pearson correlation and similar magnitude of the SOS parameter values for both tests.

In the last study, we considered the assessment of the overall quality of a HAS session. This test was conducted to assess the applicability of the proposed centre-crop approach for quality assessment considering longer-duration videos ($\ge 1\,min$). For this, we used videos of $2\,min$ duration from the lab test conducted as part of the *P.NATS Phase 2* competition. In contrast to the short-term and image quality tests, the overall content may be more important for long-term videos, therefore we increased the centre crop of the finally to-be-rated stimuli to 720*p* for the online test to maintain an appropriate level of interest in the participants. Furthermore, in this test, the participants were recruited using the Clickworkers platform and were paid. A total of 100 subjects participated in the study and the comparison results between the lab and crowd tests showed similar results on reliability and similarity as for the image and short-term video quality studies.

Overall, from these three studies, it can be concluded that the proposed test methodology for conducting quality assessment studies containing high-resolution images and videos works well for the considered task. In addition, the analysis of the online tests and the comparison with corresponding lab tests showed high reliability of the online tests. With this, it can be concluded that the defined research questions have been tackled with sufficient rigour. In future work, as a first step, a more detailed analysis of the pre-test questionnaire will be considered to investigate the impact of the factors covered in the pre-test questionnaire on the quality ratings. As for future tests, further checks based on the guidelines by Hossfeld et al. [36] will be incorporated. Furthermore, corresponding tests using the proposed methodology for the three different use cases considered in this paper will be conducted in traditional lab settings and a comparison of the results with the online studies will be performed. Future work can also include the extension of the centre- or patch-based approach to include saliency or region of interest estimations.

## Footnotes

1 https://jpegai.github.io/.

2 https://github.com/TheFragebogen/TheFragebogen.

3 https://github.com/ldvpublic/QualityCrowd2.

4 https://www.adobe.com/products/flashplayer/end-of-life.html.

5 https://github.com/NTIA/WEST.

6 http://vqegjeg.intec.ugent.be/wiki/index.php/VQEGplayer-main.

7 https://github.com/Telecommunication-Telemedia-Assessment/AVRate.

8 https://github.com/Telecommunication-Telemedia-Assessment/avrateNG.

9 https://mpv.io/.

10 https://ffmpeg.org/.

11 https://github.com/Telecommunication-Telemedia-Assessment/AVTrack360/.

12 https://github.com/audiolabs/webMUSHRA.

13 https://github.com/Telecommunication-Telemedia-Assessment/AVrateVoyager.

14 https://github.com/Telecommunication-Telemedia-Assessment/image_quality_test.

15 Using the Python implementation from https://github.com/Telecommunication-Telemedia-Assessment/SITI.

16 https://mpv.io/.

17 https://www.clickworker.com/.
